# A Smart Core-Crosslinked Supramolecular Drug Delivery System (SDDS) Enabled by Pendant Cyclodextrins Encapsulation of Drug Dimers via Host-Guest Interaction

**DOI:** 10.3390/bios11090306

**Published:** 2021-08-30

**Authors:** Xin Xing, Zhijun Guo, Yue Su, Zhen Yang, Jiwen Qian, Chunlai Tu, Lijuan Zhu, Feng Xu

**Affiliations:** 1 Department of Pharmacy and Central Laboratory, Fengxian Hospital, Southern Medical University, 6600 Nanfeng Road, Shanghai 201499, China; xingxin_01@live.cn (X.X.); junzizhuzhu@163.com (Z.G.); YZ910402@smu.edu.cn (Z.Y.); 2 Frontiers Science Center for Transformative Molecules, School of Chemistry and Chemical Engineering, Shanghai Jiao Tong University, 800 Dongchuan Road, Shanghai 200240, China; yuesu@sjtu.edu.cn (Y.S.); qjw@sjtu.edu.cn (J.Q.); zjtcl@sjtu.edu.cn (C.T.); 3 Institute of Molecular Medicine, Renji Hospital, School of Medicine, Shanghai Jiao Tong University, 160 Pujian Road, Shanghai 200217, China

**Keywords:** cyclodextrin-based polymer, core-crosslinked micelles, drug dimer, supramolecular drug delivery system, stimuli-responsive

## Abstract

Owing to poor aqueous solubility and low delivery efficiency, most of anti-cancer chemodrugs depend on various smart drug delivery platforms to enhance the treatment efficacy. Herein, a stimuli-responsive supramolecular drug delivery system (SDDS) is developed based on polymeric cyclodextrins (PCD) which crosslinked by stimuli-cleavable drug dimers via host-guest interaction. PEGylated PCD was precisely controlled synthesized by ring-opening polymerization and azide-alkyne click chemistry, and two doxorubicins (DOX) were linked with a disulfide bond to form a drug dimer (ss-DOX). They then co-assembled into supramolecular micelles. Drug dimers were utilized as cross-linkers to stabilize the micelles. The drug loading efficiency was very high that could be up to 98%. The size and morphology were measured by DLS and TEM. Owing to the disulfide bonds of drug dimers, these supramolecular micelles were dissociated by treating with dithiothreitol (DTT). In the meanwhile, the free DOXs were recovered and released from cavities of cyclodextrins because of dynamic equilibrium and hydrophilicity changes. The release profile was studied under mimic physiological conditions. Furthermore, in vitro cytotoxicity study showed excellent anti-cancer efficacy of reduced-responsive supramolecular polymeric micelles. Therefore, it can be served as a safe and stimuli-responsive SDDS for cancer therapy.

## 1. Introduction

Malignant tumors seriously jeopardize the health of the human. Among different treatment options, chemotherapy is still the most widespread strategy in clinical application [1,2,3,4]. With the development of personalized medicine, the drawbacks of traditional anti-cancer chemodrugs strongly hinder their further performance. Therefore, smart and safe drug delivery systems, which will endow the drugs with properties of long circulating time, controlled release, targeted delivery and so on, have gained significant attentions over the past decade [5,6,7,8,9]. Biodegradable and biocompatible polymeric carriers are widely used due to their designable structures and biosafety. Such as biodegradable polyesters [10,11,12], including poly(glycolic acid) (PGA) [13] and poly(ε-caprolactone) (PCL) [14], are widely applied in drug delivery and tissue engineering. Besides, poly(α-hydroxyl acid) (PHA), derived from amine acids and controlled synthesized from ring-opening polymerization, is an emerging polyester [15,16]. PHA with controlled molecular weight and narrow polydispersity index (PDI) will be a promising candidate biomaterial for clinical application.

As potential drug delivery carriers, kinds of polyesters usually conjugate with polyethylene glycol (PEG) via different synthetic routes to endow with amphiphilicity. Traditionally, amphiphilic PEG-Polyesters self-assemble into polymeric micelles, and thus chemodrugs can be easily incorporated into hydrophobic domains. However, the drug-loading efficiencies of these polymeric drug delivery systems (PDDS) are still low for hydrophobic or hydrophilic chemodrugs [5,17,18]. Moreover, chemodrugs will be burst release from unstable PDDS before they are delivered into target tumor tissue. Although trigger responsive chemistry has been used in smart polymeric micelles controlling disruption of micelles and drug release, the abovementioned shortcomings of PDDS still remain to be improved. Therefore, high drug loading efficiency and stimuli-responsive controlled drug release are two key factors for developing the novel PDDS based on polyester [19,20,21,22,23].

**Scheme 1:**
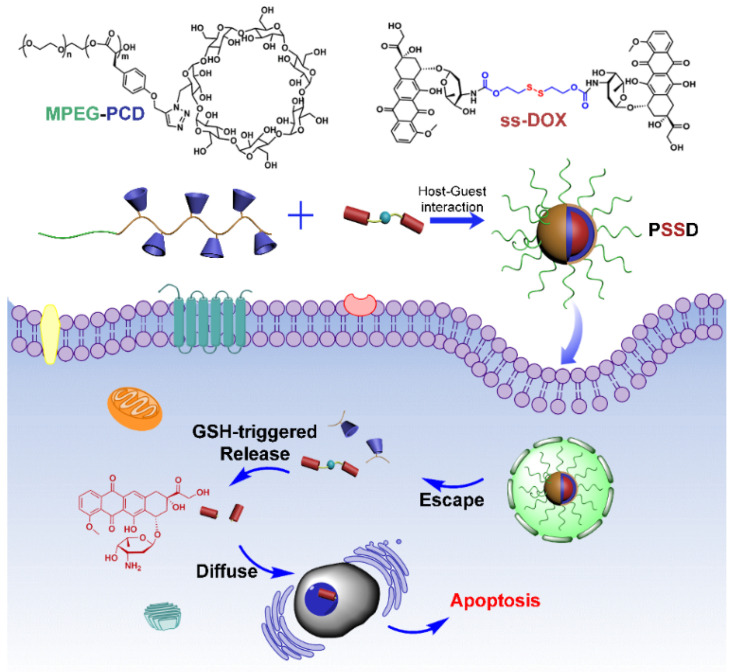
The reducible-responsive SDDS base on the drug dimer crosslinking strategy for cancer therapy.

In this study, we took the advantages of polymeric micelles and supramolecular chemistry to create a unique core-crosslinked micellar system. Herein, we constructed a double hydrophilic copolymer with pendant cyclodextrins and a drug dimer linked with disulfide bond (as shown in Scheme 1). The precise controlled copolymers, poly(ethylene glycol) methyl ether 2000-poly(α-hydroxyl acid) (MPEG-PHA), with narrow PDI were prepared by ring-opening polymerization of *O*-carboxyanhydride (OCA) monomers that initiated from poly (ethylene glycol) monomethyl ether (mPEG). The alkynyl side chain reacted with azide β-cyclodextrin (β-CD) via click chemistry. As shown in Scheme 1, MPEG-PCD and drug dimers (ss-DOX) co-assembled into supramolecular micelles in aqueous via host-guest interaction [24,25,26]. Drug dimers were utilized as the cross-linkers to stabilize the micelles. Due to the disulfide bonds, these supramolecular micelles could be dissociated by treating with reducing reagent [27,28,29]. The DOXs were exposed to aqueous environment and released from cavities of β-CDs because of dynamic equilibrium and hydrophilicity changed. The double hydrophilic polymeric carrier would be fast cleared by renal clearance after drug delivered and micelles dissociated. Benefit from the drug dimer crosslinking strategy, the drug loading efficiency could be improved easily. Therefore, this smart and biosafe SDDS based on drug dimers as core crosslinking strategy will be very promising for clinical cancer chemotherapy.

## 2. Materials and Methods

### 2.1. Chemicals

Poly(ethylene glycol) monomethyl ether 2000 (MPEG2k) was purchased from Sigma-Aldrich and used without further purification. Doxorubicin hydrochloride (DOX·HCl) was obtained from Huafeng Co (Beijing, China). Dichloromethane (DCM), hexane, tetrahydrofuran (THF) and dimethylformamide (DMF) were dried by purification columns packed with 4Å molecular sieves under a nitrogen environment. Tyr-OCA monomer and 6-mono-azide-β-cyclodextrin were synthesized according to literatures [30,31].

### 2.2. General Procedure of Preparation of Copolymer MPEG-PHA

In a glove box, Tyr-OCA (98.6 mg, 0.4 mmol, 40 eq of MPEG2K) was dissolved in DCM (2 mL), followed by the addition of MPEG2K (20 mg, 0.01 mmol) and 4-(Dimethylamino)pyridine (80 μL, 0.1 M in DCM, 0.008 mmol). After the polymerization for 4 h, the samples were transferred from the glove box and precipitated with ether (45 mL). After centrifugation and removal of upper solvent, the MPEG-PHA was obtained and dried under vacuum. (88 mg, yield 87%).

^1^H NMR (DMSO-*d_6_*, 500 MHz): δ 7.04 (d, Ar*H*), 6.82 (d, Ar*H*), 5.19 (t, alpha-*H*), 4.65 (br, -PhOC*H_2_*C≡CH), 3.48 (br, C*H_2_* of PEG), 3.44 (t, 1H, -PhOCH_2_C≡C*H*), 3.20 (-OC*H_3_*), 2.89–2.98 (dd, 2H, -C*H_2_*PhOCH_2_C≡CH).

### 2.3. Synthesis of MPEG-PCD

**Figure 1:**
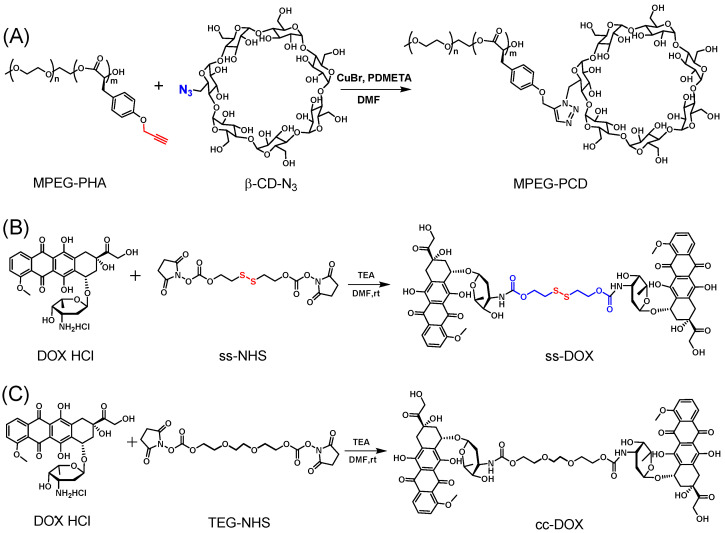
The synthesis routes of MPEG-PCD (**A**), ss-DOX (**B**) and cc-DOX (**C**).

The synthesis route of MPEG-PCD was as shown in Figure 1A. In a glove box, MPEG-PHA (88 mg, 0.35 mmol of Alkynyl) was dissolved in DMF (1 mL), followed by the addition of CuBr (5 mg, 0.035 mmol, 0.1 eq) and *N*,*N*,*N*′,*N*″,*N*″-pentamethyl-diethylenetriamine (PMDETA) (12.1 mg, 7 μL, 0.07 mmol, 0.2 eq). The suspension was stirred for 10 min and 6-mono-azide-β-cyclodextrin (450 mg, 0.38 mmol, 1.1 eq) was added. After reacted for 24 h, the samples were transferred from the glove box and precipitated in acetone (45 mL). The solid was collected by centrifuge and dissolved in water (8 mL), then precipitated in acetone (45 mL) and collected the product by centrifuge. The procedure was repeated several times until blue color disappeared. (320 mg, 59.4%).

^1^H NMR (DMSO-*d*_6_, 500 MHz): δ 8.09 (1H, triazole), 7.18 (2H, Ar*H*), 6.96 (2H, Ar*H*), 5.70 (14H, 2-O*H*, 3-O*H* of cyclodextrin), 5.29 (1H, alpha-*H*), 5.05 (2H, -PhOC*H_2_*C≡CH), 4.80 (7H, 1-*H*), 4.46 (6-O*H*), 3.94 (2H, 6-O-triazole), 3.50–3.63 (20H, 3-*H*, 5-*H*, 6-*H*) 3.48 (8H, C*H_2_* of PEG), 3.44 (t, 1H, -PhOCH_2_C≡C*H*), 3.19–3.32 (2-*H*, 4-*H*), 2.89–2.98 (2H, -C*H*_2_PhOCH_2_C≡CH).

### 2.4. Synthesis of the Cleavable DOX Dimer (ss-DOX)

The synthesis route of ss-DOX was as seen in Figure 1B. In a 5 mL vial, DOX·HCl (58 mg, 0.1 mmol) and ss-NHS (21.8 mg, 0.05 mmol) was dissolved in DMF (1 mL), and then triethylamine (16 μL, 0.12 mmol) was added. The reaction was carried at rt for 20 h and was precipitated in diethyl ether (15 mL). The red solid was collected by centrifuge and was washed by diethyl ether (10 mL) twice. The product was dried under the vacuum (62 mg, yield 96%).

^1^H NMR (DMSO-*d*_6_, 500 MHz): 7.80–7.78 (m, DOX-Ar*H* C1, C2), 7.54 (d, DOX-Ar*H* C3), 6.72 (br, C*H*-6), 5.40 (br, DOX-C*H* C1′), 5.17 (br, DOX-C*H* C7), 4.85 (t, -OC*H*_2_CH_2_SS, CH_2_-13), 4.67 (d, C*H*-5), 4.56 (d, C*H*_2_-11), 4.03 (m, C*H*-5′), 3.87 (s, C*H*_3_-4), 3.67 (br, C*H*-4′), 3.06 (m, C-10-O*H*), 2.85 (t, -OCH_2_C*H*_2_SS, C*H*_2_-14), 1.11 (d, C*H*-6′).

### 2.5. Synthesis of the Uncleavable DOX Dimer (cc-DOX)

The synthesis route of cc-DOX was as seen in Figure 1C. In a 5 mL vial, DOX·HCl (29 mg, 0.05 mmol) and TEG-NHS (10.9 mg, 0.025 mmol) was dissolved in DMF (1 mL), and then triethylamine (8 μL, 0.06 mmol) was added. The reaction was carried at rt for 20 h and was precipitated in diethyl ether (15 mL). The red solid was collected by centrifuge and was washed by diethyl ether (10 mL) twice. The product was dried under the vacuum. (28 mg, yield 86.7%).

^1^H NMR (DMSO-*d*_6_, 500 MHz): 7.83–7.78 (m, DOX-Ar*H* C1, C2), 7.56 (d, DOX-Ar*H* C3), 6.74 (br, C*H*-6), 5.40 (br, DOX-C*H* C1′), 5.19 (br, DOX-C*H* C7), 4.86 (t, -OC*H*_2_CH_2_SS, C*H*_2_-13), 4.65 (d, C*H*-5), 4.56 (d, C*H*_2_-11), 4.13 (m, C*H*-5′), 3.90 (s, C*H*_3_-4), 3.66 (br, C*H*-4′), 3.47–3.21 (m, -OC*H*_2_C*H*_2_O-), 3.05 (m, C-10-O*H*), 2.185 (t, C*H*_2_-8), 1.09 (d, C*H*-6′).

### 2.6. Preparation of DOX-Loaded and DOX Dimers Cross-Linked Micelles

DOX-loaded, ss-DOX, cc-DOX cross-linked micelles were prepared as follows. Firstly, DOX·HCl (4.8 mg) and two molar equivalents of triethylamine (for P-DOX) or ss-DOX (for PSSD), cc-DOX (for PCCD) were dissolved in DMF for 30 min to obtain deprotonated DOX solution. The above DOX solution was added into the MPEG-PHA solution (10 mg dissolved in 2 mL DMF). Then the mixture was stirred at room temperature for 2 h. After that, the mixture was added dropwise to deionized water (20 mL) under vigorous stirring and stirred for an additional 2 h. Finally, the solution was dialyzed against deionized water for 24 h (MWCO = 3500), during which the water was refreshed every 4 h. The total drug loading of ss-DOX cross-linked micelles were determined by UV-Vis and the details are as follows. The ss-DOX cross-linked micelles were treated with DTT solution (10 mM) for 6 h to obtain the released free DOX. Then the UV absorbance at 500 nm was measured to determine the DOX concentration. Drug loading content (DLC) and drug loading efficiency (DLE) were calculated according to the following formulas:

DLC (wt%) = (weight of loaded drug/weight of polymer) × 100%

DLE (wt%) = (weight of loaded drug/weight in feed) × 100%

### 2.7. Characterization of Materials

#### 2.7.1. Nuclear Magnetic Resonance (NMR) Experiments

^1^H NMR spectra of all synthesized samples were collected at a temperature of 298 K on a Varian U400 MHz spectrometer.

#### 2.7.2. Size Exclusion Chromatography (SEC) Measurements

Size exclusion chromatography (SEC) was used to determine the molecular weight and polydispersity index (PDI) of MPEG-PHAs. MPEG-PHAs were dissolved in DMF and performed on a system equipped with an isocratic pump, a DAWN HELEOS multi-angle laser light scattering detector (MALLS) detector, and an Optilab Rex refractive index detector. The MWs of polymers were determined based on the dn/dc value of each polymer sample calculated offline by using the internal calibration system processed by the ASTRA V software (Wyatt Technology, Santa Barbara, CA, USA).

#### 2.7.3. Study of the Stoichiometry of the Complex

The stoichiometry of complex of ss-DOX with β-CD in DI water/DMF (19:1) was determined by the continuous variation method, analyzing the change in fluorescent intensity of ss-DOX, where the total concentration of ss-DOX and β-CD were kept at 50 μM. Stoichiometry of the complex was calculated according to the following formula.

χ = [β-CD]/([β-CD] + [ss-DOX])

#### 2.7.4. Drug Release Experiments

The drug release profiled were performed at 37 °C in both mimetic physiological and lysosome conditions: pH = 7.4 and 5.3 buffer solutions with and without DTT (10 mM). 0.5 mg DOX-loaded MPEG-PHA micelles (P-DOX) or ss-DOX cross-linked MPEG-PHA micelles (PSSD) were dissolved in 1mL different buffer solutions and placed in a dialysis bag with a molecular weight cut-off (MWCO) of 3.5 kDa. Then the dialysis bag was immersed in 30 mL of the different release medium and incubated at 37 °C. Samples (2 mL) were periodically sucked up at different time intervals. Meanwhile, the same volume of fresh medium was filled. The amount of released DOX was then analyzed with UV/Vis spectroscopy (Lambda20, Perkin Elmer, Inc., Waltham, MA, USA) at 500 nm. The drug release studies were performed in triplicate for each of the samples.

#### 2.7.5. Dynamic Light Scattering (DLS) Measurements

The concentration of P-DOX and PSSD in these aqueous solutions are: 0.4 mg/mL. Average hydrodynamic diameters were recorded on Zetasizer Nano S (Malvern Panalytical Ltd., Malvern, UK).

#### 2.7.6. Transmission Electron Microscopy (TEM)

TEM studies were performed with a JEOL 2010 instrument operated at 200 kV. The samples of PCCD and PSSD were treated with DTT (10 mM). DTT (1.5 mg) was dissolved in PCCD and PSSD solution stirred for 2 h. A drop of PCCD/PSSD with or without DTT treated aqueous solution was added onto the surface of grid and stayed for 30 min. The excess water was sucked by filter paper from the edge and negative stained by Uranylacetate (2%, *w*/*w*) aqueous solution for 1 min and removed by filter paper.

#### 2.7.7. Confocal Laser Scanning Microscopy (CLSM)

The cellular uptake of DOX-loaded PD micelles (P-DOX), PCCD and PSSD were analyzed by CLSM. HeLa cells were seeded in six-well plates with a cell density of 2 × 10^5^ cells per well in 6-well plate and cultured in incubator at 37 °C overnight. After that, the culture solutions were replaced by free DOX, DOX-loaded PD micelles (P-DOX), PCCD and PSSD DMEM solutions at a final DOX concentration of 10 μg/mL and incubated for additional 0.5 h and 2 h. Then, the culture medium was removed and cells were washed with PBS and fixed with 4% formaldehyde for 30 min at room temperature. Finally, the cells were rinsed with PBS and stained with 2-(4-amidinophenyl)-6-indole-carbamidine dihydrochloride (DAPI) (2 μg/mL) for 10 min. The slides were mounted and observed by an LSM 700.

#### 2.7.8. Flow Cytometric Analysis

HeLa cells were seeded on 12-well plates at 1 × 10^5^ cells/well and cultured for 24 h at 37 °C. Then the culture medium was replaced by DMEM (500 μL) into which DOX loaded micelles (PCCD, PSSD) or free DOX were added (10 μg DOX/mL). After incubation at 37 °C for another 1 h, cells were washed with cold PBS for three times before collected and then subjected to flow cytometry analysis. Cells without DOX treatment served as the negative control. 

#### 2.7.9. In Vitro Anticancer Experiments

The in vitro anticancer effect of DOX-loaded PD micelles (P-DOX), PCCD and PSSD were measured by (3-(4,5-dimethylthiazol-2-yl)-2,5-diphenyl tetrazolium bromide) assay (MTT assay). HeLa cells with a density of 4.0 × 10^3^ cells/well were seeded into a 96-well plate and cultured in incubator for 24 h. Then the incubated medium was replaced by treated with GSH (10 mM in DMEM) and incubated for another 12 h using without pretreatment cells as the control. After the GSH was removed, PSSD diluted in DMEM (100 μL) were added to cells and cultured for additional 48 h. The culture medium in control were removed and replaced with 100 μL fresh medium containing serial dilutions of free DOX, PCCD and PSSD respectively. Thereafter, 15 μL of 5 mg/mL MTT assays stock solution in PBS was added to each well and incubated for another 4 h. After discarding the culture medium, the obtained purple formazan crystals were dissolved in 100 μL per well DMSO and the absorbance was measured at a wavelength of 570 nm.

## 3. Results and Discussion

### 3.1. Synthesis and Characterization of Copolymer MPEG-PCD and DOX Dimers

**Figure 2:**
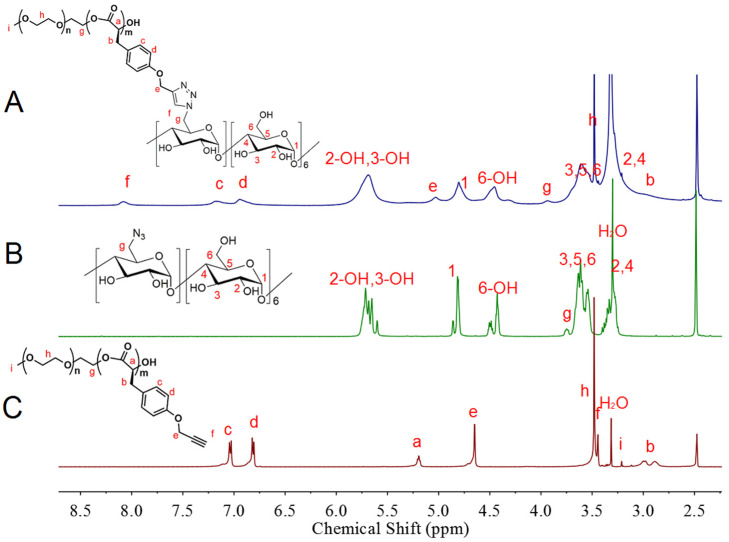
^1^H NMR spectra of (**A**) MPEG-PCD; (**B**) CD-N3 and (**C**) MPEG-PHA.

The polymerization of Tyr-OCA was initiated from hydroxyl group of MPEG2K. After the purification of copolymer, alkynyl pendant groups of the copolymer were reacted with azide groups of 6-mono-azide-cyclodextrin via click chemistry. The products were analyzed by ^1^H NMR technique (Figure 2). Besides the characteristic peaks of triazole at 8.09 ppm, the peaks at 5.05 ppm and 3.94 ppm belong to the methylene protons of e and g shifted obviously. These results confirm the formation of the triazole ring. The integral area of triazole compared to that of phenol in tyrosine is 0.25, which means all of alkynyl groups were modified with cyclodextrins. Therefore, a double hydrophilic MPEG-PCD was successfully synthesized.

The drug dimer (ss-DOX) was conjugated with a disulfide bond and served as a prodrug. After the cleavage of disulfide bond, the free DOX would recover from the drug dimer. There are one amine group and three hydroxyl groups in the structure of DOX. Highly selective modification of DOX is necessary. Firstly, we synthesized active ester by reaction of dithiodiethanol with phosgene and NHS. NHS group reacted with primary amine readily. Proton signal at 7.93 ppm belongs to amine groups disappeared. A new set of triple peaks at 4.85 ppm and 2.85 ppm belongs to methylene of dithiodiethanol appeared. Secondly, as a control, uncleavable linked drug dimer (cc-DOX) was prepared by conjugation of DOX and NHS-TEG. The yields of the final products were very high and clearly characterized by NMR.

### 3.2. Physical Properties of MPEG-PHA, MPEG-PCD, PSSD and PCCD

Weight-average molecular weights (Mw) and polydispersity index (PDI) of MPEG-PHA were determined by the SEC-MALLS technique. The Molecular Weight of this batch of MPEG-PHA is Mw = 9.7 × 10^3^ with a PDI value of 1.15. These results coincide with the calculated Mw. Due to the large number of hydroxyl groups of cyclodextrin, Mw of MPEG-PCD can’t be detected in DMF.

**Figure 3:**
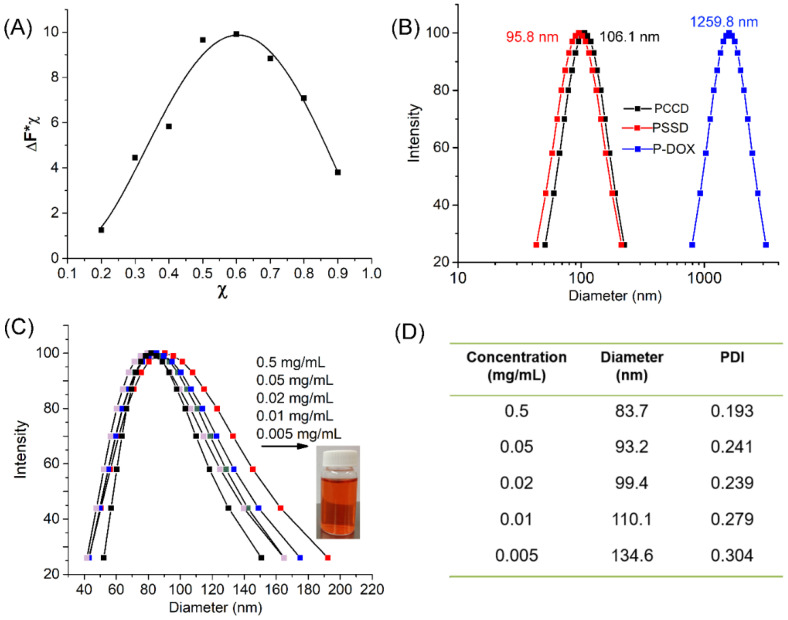
(**A**) Job’s plot of ss-DOX with β-CD; (**B**) Hydrodynamic diameters of P-DOX, PCCD and PSSD in aqueous solution measured by DLS at 37 °C; (**C**) DLS results of PSSD which was diluted to different concentrations. Inline image is the photograph PSSD solution of 0.5 mg/mL; (**D**) Diameters and Size distribution data of diluted PSSD solutions.

Cyclodextrin has a hydrophobic cavity. In an aqueous solution, cyclodextrins were able to encapsulate drug dimers and spontaneously self-assemble into micelles. The stoichiometry of the complex of ss-DOX and β-CD is an important parameter in supramolecular chemistry and will guild for drug loading. It was determined by the continuous variation method and analyzing the change in fluorescent intensity of encapsulated β-DOX. The curve in Figure 3A indicated that fluorescent intensity changes (ΔF) was varied with different ratio of β-CD and ss-DOX. By calculated with the formula χ = [β-CD]/([β-CD] + [ss-DOX]), the maximize value is at χ = 0.6 that means one DOX were encapsulated by two β-CDs. As shown in Figure 3B, the mean diameter of P-DOX, PCCD and PSSD was 1259.8 ± 69.7 nm (PDI = 0.241), 106.1 ± 0.5 nm (PDI = 0.236) and 95.8 ± 1.8 nm (PDI = 0.262), respectively, while there was no signal detected in P-CD solution due to its double hydrophilic property. These results indicate that PSSD and PCCD have formed uniform nanosized micelles and their size below 200 nm are favorable for passive targeting delivery via the EPR effect [32]. However, P-DOX solution was a little bit turbid when the hydrophobic DOXs were encapsulated in the CD. The MPEG-PCD@DOX seemed to be an amphiphilic system and it was not very stable in aqueous solution after aggregation.

The structural stability of micelles is very important during the drug delivery. The non-crosslinked micelles would be dissociated under critical micelle concentration (CMC), while the core-crosslinked micelles would be structurally stable upon dilution. To demonstrate the micelles whether they would be dissociated at very low concentration, different PSSD solutions were measured by DLS. The lognormal size distribution results were shown in Figure 3C and the corresponding data were list in Figure 3D, the diameters of PSSD micelles were kept below 150 nm on even the concentration was diluted to 0.005 mg/mL. The PSSD solution at the concentration of 0.5 mg/mL was red and transparent (insert in Figure 3C). The diameter changed from 83.7 nm to 134.6 nm when the concentration was diluted 100 times. Moreover, the PDI of diluted PSSD was still very narrow. It is speculated that MPEG-PCD was a double hydrophilic polymer, which was different from amphiphilic block polymers and had no CMC. When it was crosslinked by drug dimers, it would form stable core-crosslinked micelle. It is facilitated for PSSD intravenous.

**Figure 4:**
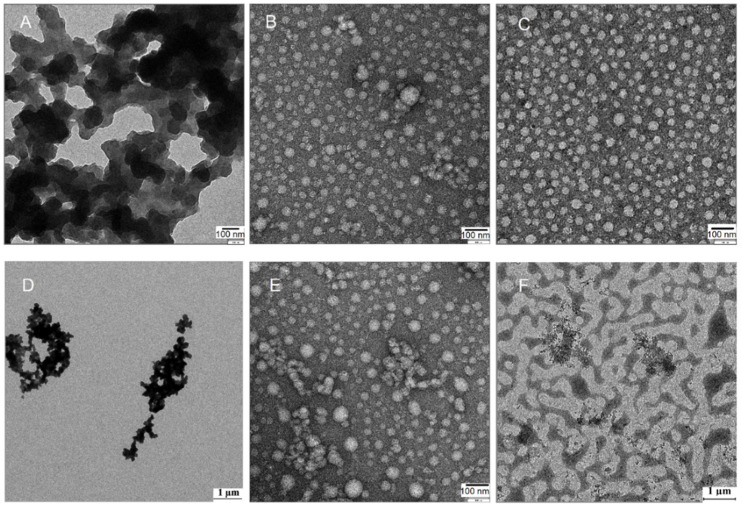
TEM images of P-DOX, PCCD and PSSD before (**A**: P-CD, **B**: PCCD, **C**: PSSD) and (**D**: PCD, **E**: PCCD, **F**: PSSD) after treatment of DTT (10 mM) for 2 h.

To observe the sizes and morphologies of P-DOX, PCCD and PSSD, TEM was applied. In an aqueous solution, pendent CDs of copolymer were able to encapsulate the free DOX, cc-DOX, ss-DOX and spontaneously self-assemble into micelles. The TEM images were shown in Figure 4A–C. P-DOX was aggregated, while a lot of spherical micelles were found in the images of PSSD and PCCD samples. The mean diameters of PSSD and PCCD were both about 50 nm which were less than that measured by DLS due to the dry effect. These TEM images indicate the formation of nanosized supramolecular micelles through the drug dimers cross-linking the core. Furthermore, to evaluate the redox-responsive behavior, three micellar solutions were treated with DTT solution. As seen in Figure 4D–F, there were no obvious changes of the morphologies in P-DOX and PCCD samples after DTT was added for 2 h (Figure 4D,E), while spherical micelles of PSSD disappeared and changed to be amorphous (Figure 4F). It indicates that the micelles of PSSD were dissociated. Because the disulfide bonds of ss-DOXs were stimuli-responsive cleavage under the reducing condition. This PSSD supramolecular micelle would be served as a stimuli-responsive drug delivery system which could controlled release free DOX after endocytosis.

### 3.3. Drug Release Behavior

It is well-known that drug-loading efficiency is significant. Drug-loading content and drug-loading efficiency of ss-DOX cross-linked micelles were measured by UV/Vis spectroscopy. Based on the standard curve of DOX at 500 nm, DOX loading was 45.7% and loading efficiency was high up to 99% in PSSD while free DOX loaded micelles lost after dialysis and drug loading efficiency was only 26.7%. The high drug loading content and drug loading efficiency were attributed to the host-guest and hydrophobic- hydrophobic interactions. 

To further access the stimuli-responsive property of ss-DOX micelles, the drug release behavior of PSSD at different pH values (simulated physiological condition, PBS, pH = 7.4 and an acidic environment, PBS, pH = 5.5) and with or without DTT (10 mM) was observed. The P-DOX was measured as a control and only evaluated under different pH values (pH = 7.4 and pH = 5.5).

**Figure 5:**
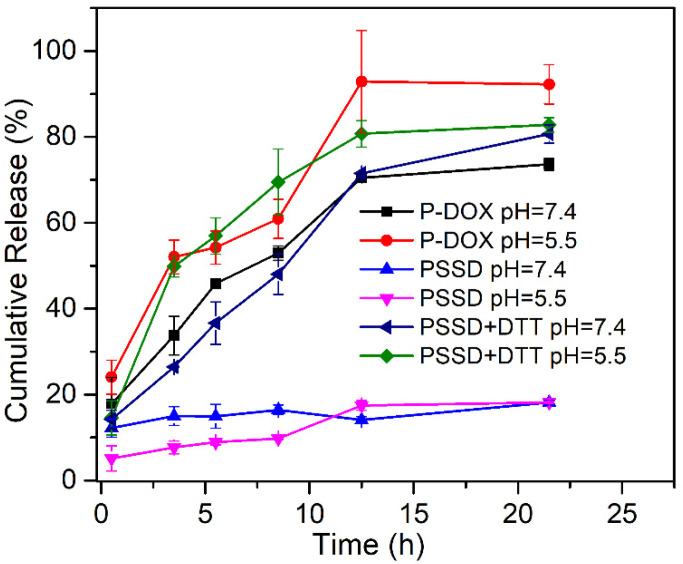
Cumulative release curves of DOX from P-DOX and PSSD with or without DTT (10 mM) at 37 °C with different pH values: pH = 7.4 PBS and pH = 5.5 acetate buffer solutions, respectively.

The drug release curves in Figure 5 show that DOXs were rapidly released from P-DOX, especially under the acidic condition. However, DTT strongly influenced the DOX release from the PSSD micelles. The final cumulative drug release without DTT was below 20% after over 20 h, suggesting that PSSD micelles were stable. However, an initial 70% release arrived only after 12 h with DTT, and the cumulative release reached a plateau of almost 70% after 20 h. These results show that the release profile of PSSD micelles had considerable DTT dependence. The disulfide linkages would undergo cleavage under reducing condition and induced the dissociation of micelles, resulting in the release of drug. DTT strongly influenced the DOX release from the PSSD micelles. Release profile of PSSD micelles were not pH-dependence. DOX was not released completely, it might exist the dynamic equilibrium between β-CD and DOX even it was at low concentration.

**Figure 6:**
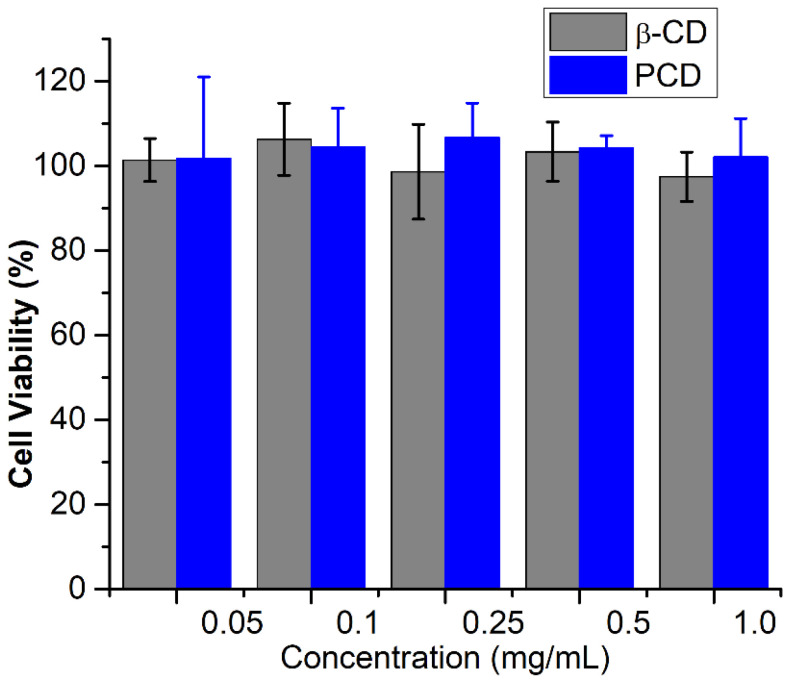
Cell viability of P-CD against HeLa cells after incubated for 24 h with different formulations concentration. HeLa cells incubated without micelles were used as the control.

PHA is a biodegradable polyester which derives from tyrosine. Meanwhile, β-CD is also a natural product. Cytotoxicity of PCD in which PHA conjugated with β-CD was assessed by MTT assay. As confirmed in Figure 6, cell viability of β-CD or PCD is almost 100% even at high concentration (1 mg/mL). It suggests that PCD has no cytotoxicity and is suit for a drug delivery carrier.

**Figure 7:**
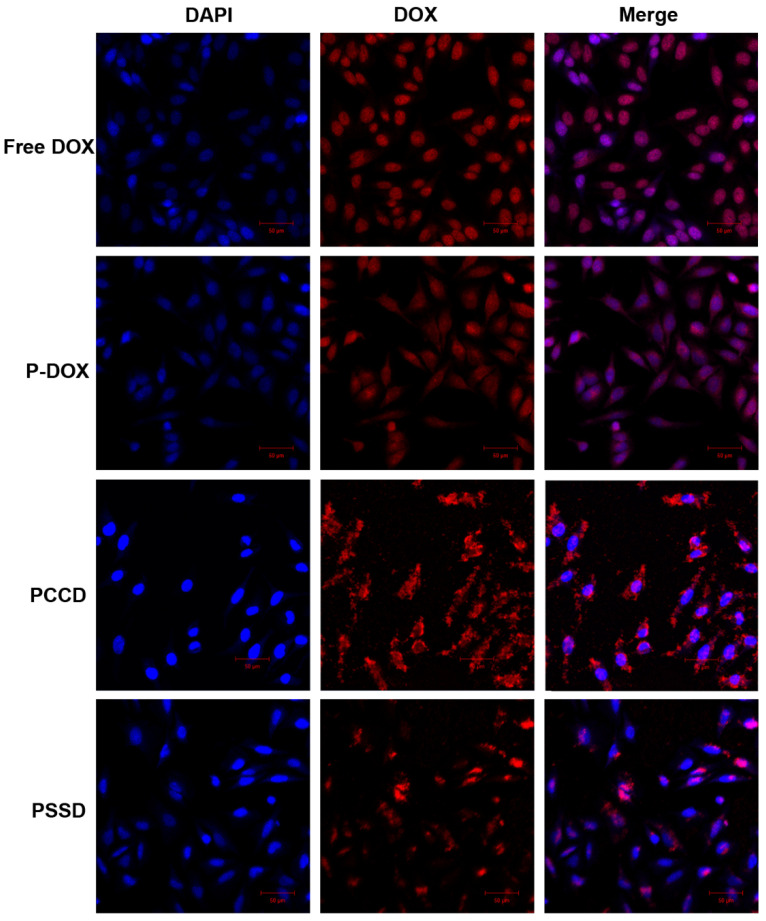
CLSM images of HeLa cells incubated with free DOX, P-DOX, PCCD and PSSD (red) for 2 h respectively. Cell nuclei were stained with DAPI (blue).

CLSM was used to evaluate the cellular uptake profile of DOX, P-DOX, PCCD and PSSD. HeLa cells were incubated with different formulations at 37 °C for different time intervals, 0.5 h and 2 h, respectively, and then fixed with paraformaldehyde. After that, the treated samples were observed directly with CLSM in the fluorescent mode. As shown in Figure 7, free DOX treated HeLa cells exhibited strong red fluorescence in the nucleus after 2 h incubation. For P-DOX treated HeLa cells, strong red fluorescence of DOX was observed both in cytoplasm and nuclei, implying that part of DOX released from P-DOX after 2 h incubation. However, the red fluorescence of DOX were only shown in cytoplasm for PCCD and PSSD treated HeLa cells, demonstrating the controlled release of DOX in PSSD.

**Figure 8:**
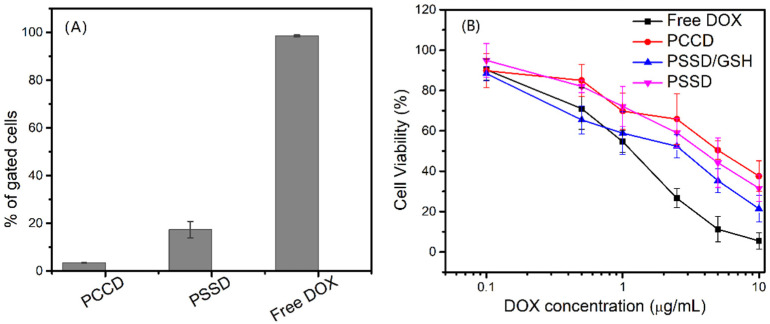
(**A**) Flow cytometric analyses of HeLa cells after 1 h incubation with PCCD, PSSD and free DOX (n = 3) at 37 °C. (**B**) Cell viability of HeLa cells incubated with PSSD without (▼) or with (▲) GSH-Oet (10 mM), PCCD (●) and free DOX (■) at different drug concentrations for 48 h. Non-pretreated group was used as a control. Data are presented as the average standard deviation (n = 5).

Flow cytometry was further conducted to quantitatively evaluate the cellular uptake level of DOX dimer cross-linked micelles (PCCD and PSSD). As shown in Figure 8A, only 3.45% of PCCD and 17.34% of PSSD compared to free DOX (100%) were entranced into HeLa cancer cells after 1 h at 37 °C, which indicated that the endocytosis efficiency of PSSD or PCCD was less than that of free DOX.

MTT assay was performed to evaluate the in vitro anticancer efficacy of PSSD in HeLa cells. As shown in Figure 8B, the half maximal inhibitory concentration (IC_50_) of PSSD/GSH against HeLa cells (2.5 μg/mL) is lower than that of PSSD (4 μg/mL) and PCCD (6 μg/mL), indicating the reduction responsive drug release. Meanwhile, the IC_50_ value of free DOX against HeLa cells (1.2 μg/mL) was lowest in all treated group, which was attributed to the much quicker in vitro cellular uptake of free DOX than that of nanoparticles.

## 4. Conclusions

A biocompatible poly(α-hydroxyl acid) (PHA) bearing with pendant β-cyclodextrins and two drug dimers are successfully synthesized and systematically characterized. Moreover, a smart SDDS is developed based on host-guest interaction in which reducible-cleaved drug dimers (ss-DOX) are served as core cross-linkers. They can self-assemble into spherical nanosized micelles. These uniform supramolecular micelles are stable in aqueous solution benefit from core cross-linking and PEGylation, while it dissociates and trigger releases free DOXs under the reducing condition. The drug loading efficiency of drug dimers cross-linked micelles is almost quantitative. It is much higher than free DOX loaded micelles. PSSD will be endocytosed into the cytoplasm and the nucleus. The anti-cancer efficacy of PSSD is obvious improved when the HeLa cells treat with GSH. This strategy will be extended to various anti-cancer chemodrugs. MPEG-PCD can be served as a smart platform for development of SDDS with controlled release and long circulation.

## Data Availability

The data that support the findings of this study are available on request from the corresponding author.
